# Data on risk factors related to in-hospital mortality in patients less than 55 years of age with ST-segment elevation myocardial infarction

**DOI:** 10.1016/j.dib.2022.108084

**Published:** 2022-03-23

**Authors:** Marta Lorente-Ros, Amisha Patel, José A. Lorente, Esteban López-de-Sá

**Affiliations:** a Mount Sinai Morningside, The Icahn School of Medicine at Mount Sinai, 1111 Amsterdam Ave, New York, NY 10025, United States; b Hospital Universitario de Getafe, Carr. Toledo, 12500, Madrid 28905, Spain; c CIBER de Enfermedades Respiratorias, C. de Melchor Fernández Almagro, 3, Madrid 28029, Spain; d Universidad Europea, C. Tajo, Madrid 28670, Spain; e Hospital Universitario La Paz, IdiPaz, Paseo de la Castellana, 261, Madrid, Spain; f CIBER de Enfermedades Cardiovasculares, C. de Melchor Fernández Almagro, 3, Madrid 28029, Spain

**Keywords:** Acute myocardial infarction, St-segment elevation, Young, Mortality

## Abstract

Risk factors associated with in-hospital mortality in young patients with ST-segment elevation myocardial infarction are poorly described. In addition, it is increasingly recognized that these risk factors might differ from those of older patients. The dataset herein presented describes the association between different variables and in-hospital mortality in patients <55 years old with ST-segment elevation myocardial infarction. This data supplements the manuscript “Sex Related Differences in the Treatment of ST-Segment Elevation Acute Myocardial Infarction in Patients Aged <55 Years” (Lorente-Ros et al.) Data for this data in brief article were obtained from a prospective database of patients <55 years old with confirmed ST-segment elevation myocardial infarction admitted to a tertiary care hospital during an 11-year period. The data were collected via review of the clinical charts. The dataset describes the relative risk and 95% confidence interval of in-hospital mortality for each variable, including cardiovascular risk factors, angiographic findings, treatment received, and complications developed. Patients in this dataset represent a unique population, given that it only includes confirmed ST-segment elevation myocardial infarction while excluding other types of acute coronary syndrome, the patient's young age, and the reflection of contemporary up-to-date practices. This dataset will be valuable to further build on knowledge on the prognostic markers of acute myocardial infarction in a younger patient population.

## Specifications Table

**Table:** 

Subject	Cardiology and Cardiovascular Medicine
Specific subject area	Ischemic heart disease in special populations; Mortality of ST-segment elevation acute myocardial infarction in the young
Type of data	Table
How the data were acquired	Data were acquired from a prospective database of patients admitted with confirmed ST-segment elevation myocardial infarction in a tertiary care hospital. Data was analyzed with SPSS v.22.0 (Chicago, Illinois).
Data format	Raw and analyzed
Description of data collection	All patients admitted to the Acute Cardiac Care Unit from a tertiary care hospital between 2007 and 2017 with a confirmed diagnosis of ST-segment elevation myocardial infarction were included. Patients with age ≥55 years were excluded. Patients with myocardial infarction in the context of surgery, trauma, or interventional procedures were excluded. Patients with prior ST-segment elevation myocardial infarction, and prior surgical or percutaneous revascularization were excluded.
Data source location	• Institution: *Hospital Universitario La Paz* • City/Town/Region: Madrid • Country: Spain
Data accessibility	Analyzed data: within the article. Raw data: Available upon author request to the following email: marta.lorenteros@mountsinai.org.
Related research article	M. Lorente-Ros, A. Patel, J.A. Lorente, E. López-de-Sá, Sex Related Differences in the Treatment of ST-Segment Elevation Acute Myocardial Infarction in Patients Aged <55 Years, Am. J. Cardiol. (2022): S0002–9149(22)00,076–5. 10.1016/j.amjcard.2022.01.018

## Value of the Data

• Risk factors for mortality in young patients with ST-segment elevation myocardial infarction (STEMI) are poorly described. Increasing attention needs to be focused on better defining these prognostic factors. This data in brief article analyzes risk factors associated with in-hospital mortality in these patients. The dataset represents a pure cohort of STEMI (excluding other types of acute coronary syndromes), ensuring internal validity.
• Clinical and interventional cardiologists with an interest in special populations such as the female population and the young population can use this data to gain insight on these underrepresented cohorts, as well as use it for the development of investigations which can specifically aim to study these populations in more detail.
• The herein presented data can be used for the development of future studies on the special population of young patients with STEMI. This dataset can not only provide insight for studies that focus on in-hospital mortality, but also for studies with the aim of better characterizing the clinical features of this unique population.

## 1 Data Description

This data in brief article contains information on risk factors related to in-hospital mortality in patients <55 years old with confirmed STEMI [Table 1]. The table represents each variable, including baseline characteristics, cardiovascular risk factors, angiographic findings, treatment received, and complications developed, and its association with mortality. Continuous variables are presented as mean ± standard error of the mean. Variables that are not normally distributed are described as medians and interquartile ranges. Qualitative variables are expressed as n and percetange. The strength of the associations is expressed by the relative risk and 95% confidence interval.

**Table 1:** Risk factors related to in-hospital mortality in young patients with STEMI.^

		n	Mortality n (%)	p value	RR	95% CI
**Variable**						
Female sex	+	100	2 (2.0%)	0.760	0.593	0.142–2.472
	–	712	24 (3.4%)			

Diabetes mellitus	+	99	6 (6.1%)	0.117	2.161	0.889–5.250
	–	713	20 (2.8%)			

Hypertension	+	244	5 (2.0%)	0.280	0.554	0.211–1.453
	–	568	21 (3.7%)			

Dyslipidemia	+	299	5 (1.7%)	0.064	0.409	0.156–1.072
	–	513	21 (4.1%)			

Smoking history	+	667	19 (2.8%)	0.293	0.590	0.253–1.377
	–	145	7 (4.8%)			

Triggering factor	+	127	4 (3.1%)	1.000	0.981	0.344–2.798
	–	685	22 (3.2%)			

Emotional trigger	+	12	0 (0.0%)	1.000	NA	
	–	800	26 (3.2%)			
Physical trigger	+	87	3 (3.4%)	0.752	1.087	0.333–3.546
	–	725	23 (3.2%)			

Q wave AMI	+	774	26 (3.4%)	0.628	NA	
	–	38	0 (0.0%)			

**Angiographic findings**						

MINOCA	+	14	1 (7.1%)	0.368	2.280	
	–	798	25 (3.1%)			

Non-obstructive pattern	+	31	2 (6.5%)	0.261	2.099	
	–	781	24 (3.1%)			

TIMI 2–3	+	247	7 (2.8%)	0.830	0.843	
	–	565	19 (3.4%)			

Multivessel disease	+	388	18 (4.6%)	0.029	2.459	
	–	424	8 (1.9%)			

Tortuosity	+	10	1 (10.0%)	0.279	3.208	
	–	802	25 (3.1%)			

**Treatment**						

PTCA	+	793	26 (3.3%)	1.000	NA	NA
	–	19	0 (0.0%)			

Stent implantation	+	750	25 (3.3%)	0.714	2.067	0.285–14.997
	–	62	1 (1.6%)			

Drug eluting stent *	+	424	14 (3.3%)	1.000	0.979	0.450–2.127
	–	326	11 (3.4%)			

More than one stent *	+	323	10 (3.1%)	0.839	0.881	0.401–1.936
	–	427	15 (3.5%)			

Inotropes	+	61	16 (26.2%)	<0.001	19.698	9.344–41.525
	–	751	10 (1.3%)			

Hypothermia	+	64	22 (34.4%)	<0.001	64.281	22.850–180.836
	–	748	4 (0.5%)			

Mechanical ventilation	+	82	25 (30.5%)	<0.001	222.561	30.554–1621.168
	–	730	1 (0.1%)			

Intraaortic balloon pump	+	36	13 (36.1%)	<0.001	21.556	10.786–43.076
	–	776	13 (1.7%)			

Temporal pacemaker	+	9	2 (22.2%)	0.031	7.435	2.059–26.854
	–	803	24 (3.0%)			

RRT	+	4	4 (100.0%)	<0.001	NA	NA
	–	808	22 (2.7%)			

**Complications**						

Spontaneous hemorrhage	+	10	4 (40.0%)	<0.001	14.582	6.147–34.588
	–	802	22 (2.7%)			

PTCA-related hemorrhage	+	26	3 (11.5%)	0.046	3.943	1.264–12.304
	–	786	23 (2.9%)			

Cardiac arrest	+	79	23 (29.1%)	<0.001	71.135	21.847 - 231.622
	–	733	3 (0.4%)			

CI, confidence interval. MINOCA, myocardial infarction with non-obstructive coronary arteries. PTCA, percutaneous transluminal coronary angioplasty. RR, risk ratio. RRT, renal replacement therapy. STEMI, ST-segment elevation myocardial infarction.

Values are n and percentage.

⁎ In patients with stent implantation (*n* = 750).

^ From the related research article (Ref. [1]).

## 2 Experimental Design, Materials and Methods

### 2.1 Study design

The data herein presented was obtained from a database of patients admitted with acute myocardial infarction (AMI) to the Acute Cardiac Care Unit of a tertiary care hospital in Madrid, Spain. The database is prospective and adds patient information at the moment of admission, which is then confirmed at the moment of discharge. The data in this article was obtained from retrospective review of the patients included in the above-mentioned database during an 11-year period who met all of the inclusion criteria and none of the exclusion criteria as described below.

### 2.2 Patient selection: inclusion and exclusion criteria

To obtain the data presented in this article, we included patients with a diagnosis of STEMI on admission and confirmed at discharge, who were <55 years old at the moment of admission, and who were admitted during an 11-year period between 2007 and 2017.

Exclusion criteria were:

‐ Patients with non-ST segment elevation myocardial infarction (based on the electrocardiogram performed at first medical contact).
‐ Patients aged 55 years or older at the moment of admission.
‐ Patients with STEMI in the context of surgery, trauma, or interventional procedures.
‐ Patients with a prior history of AMI.
‐ Patients with prior revascularization, either surgical or percutaneous.

All consecutive patients that met inclusion criteria and that did not meet any of the exclusion criteria were included.

### 2.3 Data collection

The collection of data was made via retrospective review of the clinical charts of included patients. Collection of information on baseline characteristics and cardiovascular risk factors was based on documented history and physical exam. Information from the angiography was collected via review of the angiographic films, as well as the angiography report, for the first angiography for each patient. Information on treatment received and complications developed was obtained from the clinical chart of each patient. The definitions used for each variable are described in the section below.

### 2.4 Variables

Data on the following variables were collected:

‐ Demographics: age, sex, height, weight.
‐ Cardiovascular risk factors: hypertension, diabetes mellitus, hyperlipidemia, smoking history, emotional or physical trigger for the AMI.
‐ Electrocardiogram characteristics: development of Q waves.
‐ Angiographic findings: obstructive versus non-obstructive AMI based on culprit vessel, distal flow, coronary vessel tortuosity, multivessel disease.
‐ Treatment: diagnostic angiography, percutaneous transluminal coronary angioplasty (PTCA), stent placement, type of stent, number of stents, inotropic support, intraaortic balloon, hypothermia, mechanical ventilation, renal replacement therapy, temporal pacemaker.
‐ Complications: cardiac arrest, spontaneous hemorrhage, PTCA-related hemorrhage.

### 2.5 Definitions

The following standard definitions were used for data collection:

‐ Non-obstructive angiography was defined as: (1) absence of fixed coronary stenosis ≥50%, or (2) coronary thrombosis or coronary embolism without evidence of atheroma plaque rupture.
‐ Myocardial Infarction with Non-Obstructive Coronary Arteries (MINOCA) was defined according to the most recent definition [2]: AMI according to its Fourth Universal definition [3] with non-obstructive coronary lesions on angiography (no fixed stenosis ≥50%), and with no specific alternate diagnosis for the clinical presentation.
‐ Distal flow was determined according to the TIMI grade flow classification [4].
‐ Multivessel disease was defined as significant stenosis in two or more major epicardial coronary arteries.
‐ Coronary vessel tortuosity was defined as two or more segments of the coronary arteries with three or more curvatures ≤120° measured during diastole.
‐ Spontaneous hemorrhagic complications were defined as major bleeding or minor clinically significant bleeding according to the International Society on Thrombosis and Haemostasis bleeding scale for non-surgical patients [5].
‐ PTCA-related or procedural related hemorrhage was defined as major bleeding according to the International Society on Thrombosis and Haemostasis bleeding scale for surgical patients [6].

### 2.6 Statistical analysis

Statistical analysis was performed with the SPSS v.22.0 (Chicago, Illinois). Continuous variables are presented as mean ± standard error of the mean and were compared by the Student´s t-test. Variables that are not normally distributed are described as medians and interquartile ranges, and differences were analyzed with the Kruskal-Wallis test. Normality was tested by the Smirnov-Kolmogorov test. Categorical variables were compared using the Pearson's chi-square test or Fisher exact test as appropriate. The strength of the associations was measured by the relative risk and 95% confidence interval. The relative risk for each variable represents the probability of in-hospital mortality when the studied variable is present divided by the probability of in-hospital mortality when the studied variable is not present. A relative risk of 1 implies there is no difference in the event of mortality if the exposure has or has not occurred. If the relative risk greater than 1, the event of mortality is more likely to occur if there was exposure. If the relative risk is less than one, the event of mortality is less likely to occur if there was exposure. A p value <0.05 was considered statistically significant.

### 2.7 Steps to reproduce the results of this data

To reproduce the results of this Data in Brief as well as create new results, first select the variable in which you would like to test an association with in-hospital mortality. Use the Smirnov-Kolmogorov test to see if the variable of choice is normally distributed. For continuous variables, calculate the mean and standard error of the mean (if distributed normally) or the median and the interquartile range (if not distributed normally). Use the Student T Test (if distributed normally) or the Mann-Whitney U Test (if not distributed normally). For categorical variables, describe them as “n” and percentage. Use the Pearson's chi-square test (if distributed normally) or Fisher exact test (if not distributed normally). To measure the strength of the association, calculate the relative risk.

## Ethics Statements

This study was approved by the Ethical committee of Hospital Universitario La Paz, Madrid Spain (PI-3172). The study was compliant with patient confidentiality regulations, with anonymization of all data.

## CRediT authorship contribution statement

**Marta Lorente-Ros:** Conceptualization, Methodology, Investigation, Writing – original draft, Visualization. **Amisha Patel:** Conceptualization, Writing – review & editing, Supervision. **José A. Lorente:** Methodology, Formal analysis, Resources, Writing – review & editing, Supervision. **Esteban López-de-Sá:** Methodology, Formal analysis, Resources, Supervision.

## Declaration of Competing Interest

The authors declare that they have no known competing financial interests or personal relationships that could have appeared to influence the work reported in this paper.

## Data Availability

(Original data) (Raw data uploaded as ``Supplementary material'' for peer review).
